# Supplementary material for: Structure and consistency of self-reported social contact networks in British secondary schools
Source: PLoS One. 2018 Jul 25;13(7):e0200090. doi: 10.1371/journal.pone.0200090 (PMC6059423; doi:10.1371/journal.pone.0200090)
Supplement: S1 Table — (PDF) [file pone.0200090.s001.pdf]

School	Round	Unique participants	Male participants	Female participants	Total contacts
1	1	61	26	35	352
	2	83	30	53	470
	3	92	36	56	525
	4	70	28	42	387
2	1	134	57	77	786
	2	121	52	69	699
	3	141	55	86	826
	4	85	33	52	505
3	1	91	91	0	525
	2	67	67	0	377
	3	67	67	0	381
	4	143	143	0	794
4	1	25	11	14	142
	2	26	9	17	144
	3	25	7	18	138
	4	21	7	14	122
